# The impact of the butterfly effect on human parainfluenza virus haemagglutinin-neuraminidase inhibitor design

**DOI:** 10.1038/s41598-017-04656-y

**Published:** 2017-07-03

**Authors:** Larissa Dirr, Ibrahim M. El-Deeb, Leonard M. G. Chavas, Patrice Guillon, Mark von Itzstein

**Affiliations:** 1 Institute for Glycomics, Griffith University, Gold Coast Campus, Queensland, 4222 Australia; 2 Experiments division, Synchrotron SOLEIL, Gif-sur-Yvette, France

## Abstract

Human parainfluenza viruses represent a leading cause of lower respiratory tract disease in children, with currently no available approved drug or vaccine. The viral surface glycoprotein haemagglutinin-neuraminidase (HN) represents an ideal antiviral target. Herein, we describe the first structure-based study on the rearrangement of key active site amino acid residues by an induced opening of the 216-loop, through the accommodation of appropriately functionalised neuraminic acid-based inhibitors. We discovered that the rearrangement is influenced by the degree of loop opening and is controlled by the neuraminic acid’s C-4 substituent’s size (large or small). In this study, we found that these rearrangements induce a butterfly effect of paramount importance in HN inhibitor design and define criteria for the ideal substituent size in two different categories of HN inhibitors and provide novel structural insight into the druggable viral HN protein.

## Introduction

Human parainfluenza virus (hPIV) is one of the leading causes of respiratory tract disease in infants and children^1, 2^ and is estimated to result in over 1.5 million cases per year in the United States alone^3^. Despite continuous efforts^4, 5^, there are neither specific antiviral therapy nor vaccines available against hPIV-3 to date. The viral surface glycoprotein haemagglutinin-neuraminidase (HN) represents an ideal target for the development of urgently needed antiviral agents. The viral HN protein encompasses three key functions in virus infection and spread. The hPIV HN recognizes and attaches to *N*-acetylneuraminic acid-containing glycoconjugates present on the host cell and subsequently activates the fusion machinery, facilitating infection. Upon virus replication, hPIV HN enzymatically cleaves the neuraminic acid (Neu), *N*-acetylneuraminic acid (Neu5Ac, **1**) from host cell receptors allowing viral spread to uninfected cells^3^. A number of Neu2en (**2**)-based inhibitors (*e.g*. BCX 2798, **3**)^6, 7^, as well as a novel approach that uses two well-established drugs^8^ in a combinatorial manner, have been used to target the hPIV-3 HN protein. However, none of these agents have progressed to the clinic.

Recently, we have reported^9^ the first structural investigation into the catalytic mechanism of the hPIV-3 HN protein using the 2,3-difluoro-*N*-acylneuraminic acid derivative, **4**. This study demonstrated that the protein forms a covalent adduct with the substrate as a result of a nucleophilic attack at the Neu moiety’s anomeric carbon (C-2) by the hydroxyl group of the key catalytic amino acid, Tyr530. Hence, it was verified^9^ that hPIV-3 HN can be targeted by reactive substrate-like inhibitors such as **4**.

**Figure 1:**
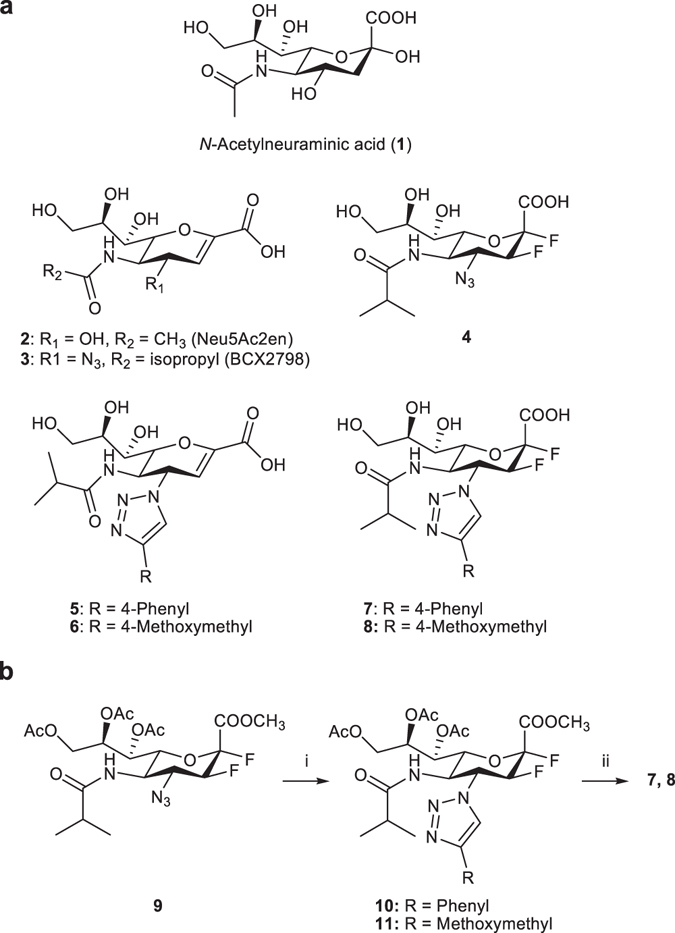
Structures of reference and novel inhibitors and synthetic strategy. (**a**) Structures of Neu5Ac (**1**), reference Neu2en hPIV-3 HN inhibitors **2, 3, 5** and **6**, the reference 2α,3β-difluoro-analogue of BCX 2798 (**4**) and the novel 2α,3β-difluoro-4-triazolo-*N*-acylneuraminic acid derivatives **7** and **8**. (**b**) (i) CuSO_4_, sodium ascorbate, MeOH/H_2_O (1:1), M.W., 80 °C, 30 min (**10**, 81%; **11**, 89%); (ii) NaOH, MeOH/H_2_O (1:1), rt, o/n (**7**, 68%; **8**, 84%).

We have also recently described the design and synthesis of novel potent 4-deoxy-4-triazolo-Neu2en-based inhibitors (**5** and **6**, Fig. 1)^10^. These inhibitors carry bulky C-4 substituents on the Neu2en template and target the proposed 216-cavity formed by movement of the flexible 216-loop^11^, a unique feature in hPIV-3 HN. Taken together these developments inspired further structural and biological investigation of Neu (**1**)-based inhibitors that incorporate a C-5 isobutyramido moiety, bulky C-4 triazole-substituents and C-2 and C-3 fluorides, such as **7** and **8**, to evaluate functional group synergism.

## Results and Discussion

### Analysis of the hPIV-3 HN structure in its open 216-loop form

To aid the development of substrate-like mechanism-based inhibitors, with further functionalization as in **5** and **6**, atomic-level details of the binding mode of both 2,3-difluoro-*N*-acylneuraminic acid-based and Neu2en-based inhibitors were required. Therefore, both inhibitors **5** and **6** were independently co-crystallized with hPIV-3 HN protein (see Supplementary Methods).

**Figure 2:**
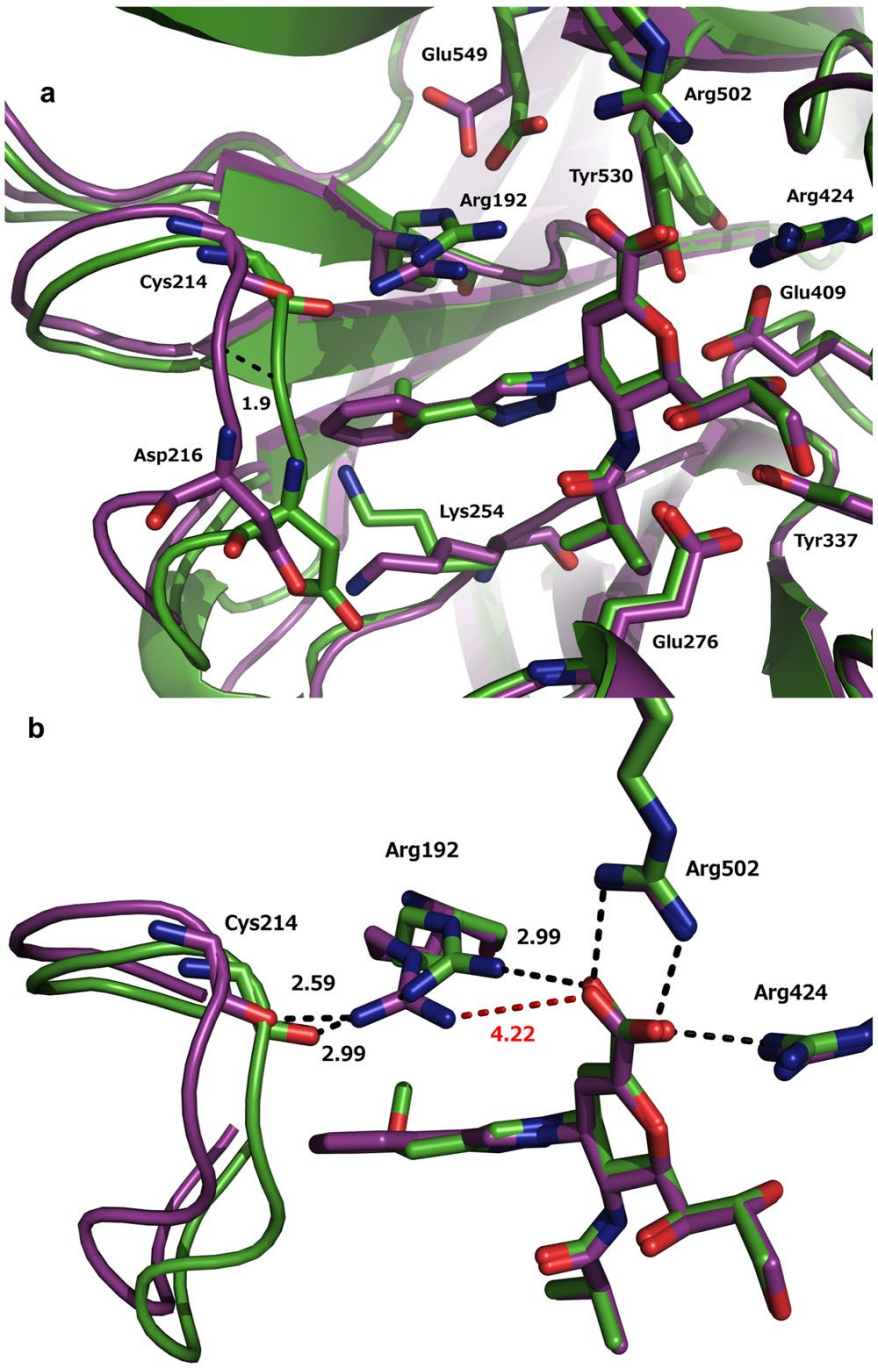
Structural insights into hPIV-3 HN–**5** and –**6** complexes. (**a**) Superimposition of the hPIV-3 HN active site with bound inhibitor **5** and bound inhibitor **6** (magenta and green, respectively). A further 1.9 Å shift in 216-loop position led to the accommodation of the bulkier 4-phenyltriazole in the hPIV-3 HN–**5** complex (dashed-line in black). (**b**) Zoom-in image displays the distances between active site residues Arg192 (side-chain) and Cys214 (backbone carbonyl oxygen) as well as the Arg192 side-chain and inhibitors **5** and **6**. Dashed-lines in black or red represent favourable (H-bond donor–acceptor distance < 4.0 Å) and unfavourable distance (H-bond donor–acceptor distance > 4.0 Å) for hydrogen bonds and salt bridge formation respectively. *O* red, *N* blue.

The crystallographic structures of hPIV-3 HN in complex with inhibitors **5** (protein data bank (PDB) accession code: 5KV9) and **6** (PDB accession code: 5KV8) demonstrated that the C-4 triazole substituents occupy the 216-cavity (Fig. 2a).

To accommodate the C-4 substituents of each inhibitor, the respective 216-loop (amino acids 210–220)^11^ was forced into a more open conformation in each complex when compared with the *apo* structure (PDB accession code: 4XJQ). In this context, the 216-loop moved, relative to the *apo* structure (measured from the protein backbone), up to 3.7 Å and 1.7 Å in the hPIV-3 HN–**5** and hPIV-3 HN–**6** complexes, respectively. To accommodate the bulkier C-4 phenyltriazole moiety associated with inhibitor **5**, the 216-loop in the hPIV-3 HN–**5** complex adopted a more open conformation in comparison to the hPIV-3 HN–**6** complex. The observed loop movement between these two complexes reached significant differences of up to 2.17 Å (*e.g*. 1.90 Å at Asp216, Fig. 2a), where the phenyltriazole moiety of **5** is directed.

### Key structural differences between hPIV-3 HN–5 and HN–6 co-crystal structures

Key differences related to the degree of loop opening were observed in the position of the triarginyl cluster and the active site residues (Tyr530, Glu549, Lys254). The triarginyl cluster is known to make substantial contribution, through engagement with the carboxylate of both substrates and inhibitors, in a broad range of neuraminidases (NAs)^9, 12–15^ including influenza A virus (IAV) NA^16–19^. A similar contribution was observed in the hPIV-3 HN–**6** complex, in which the hPIV-3 HN triarginyl cluster (comprised of arginine residues Arg192, Arg424 and Arg502) was found to form a salt bridge and hydrogen bonds with the carboxylic acid moiety of inhibitor **6** (Fig. 2b). Interestingly, a significant movement of the 216-loop, that led to a more open cavity in the hPIV-3 HN–**5** complex, was accompanied by the reorientation of Arg192. In the hPIV-3 HN–**5** complex the η2-NH_2_ of Arg192 side-chain, which usually participates in the coordination of the bound inhibitor, as in the hPIV-3 HN–**6** complex, was oriented away from its typical location by 1.23 Å (Fig. 2b). This movement enabled the formation of a hydrogen bond between Arg192 and the Cys214 backbone carbonyl oxygen (2.59 Å). However, this shift, in the position of the Arg192 side-chain (4.22 Å, Fig. 2b) prevented a hydrogen bond formation with the carboxylic acid oxygen of inhibitor **5** and loss of a crucial ionic interaction. The two other arginine residues in the triarginyl cluster, Arg424 and Arg502, were found to maintain the same orientation in both complexes and were engaged in hydrogen bonding and ionic interactions with the carboxylate moiety of both inhibitors **5** and **6**.

**Figure 3:**
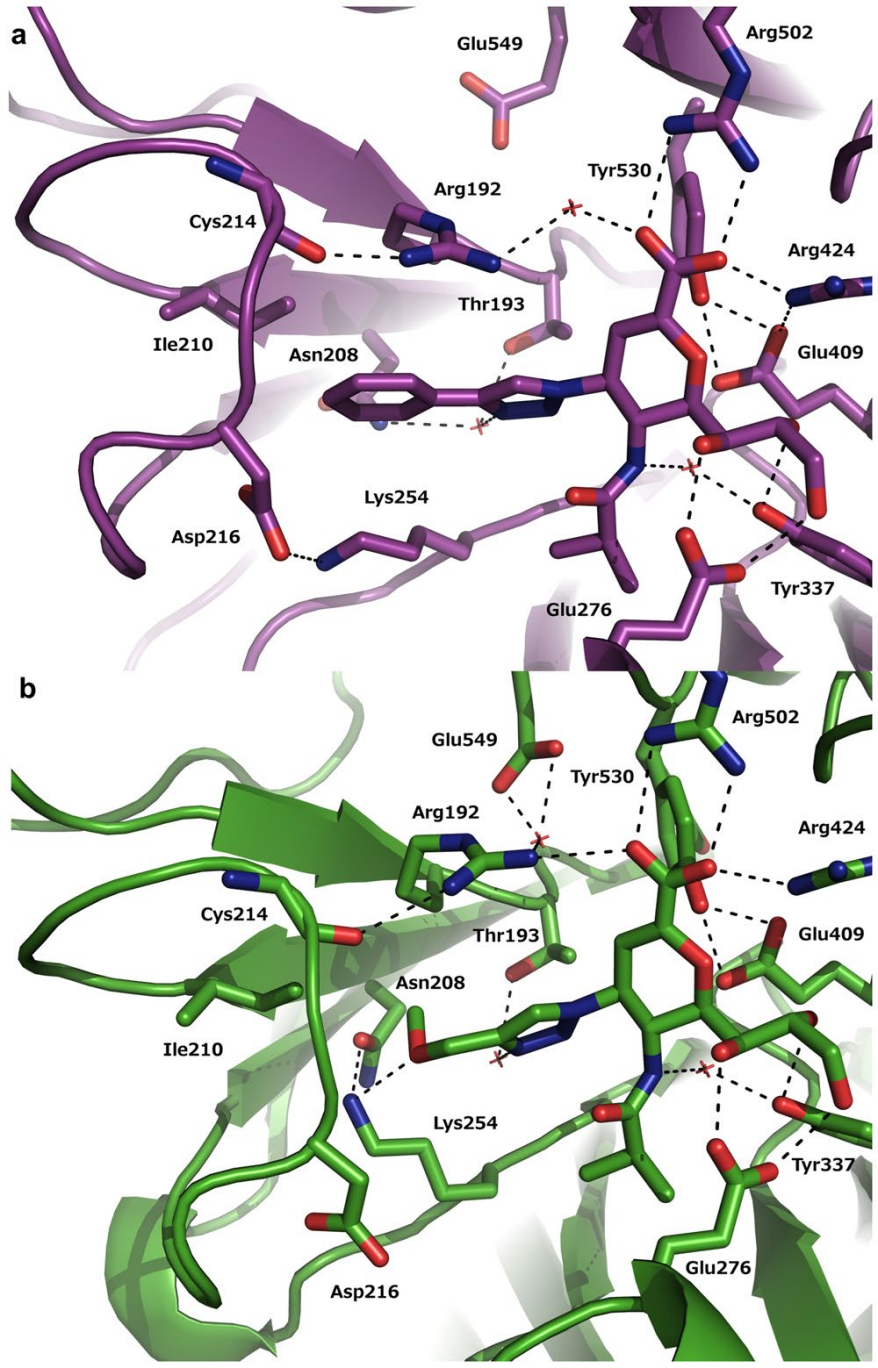
hPIV-3 HN–**5** and –**6** complexes. hPIV-3 HN active site complexed with inhibitor **5** (top) and with inhibitor **6** (bottom) displaying amino acids that are engaged in the binding event as well as three active site resident water molecules. Potential hydrogen bonds are shown as black dotted lines. *O* red, *N* blue.

Further important structural differences between the two complexes were observed for the active site residues Lys254, Tyr530 and Glu549. The C-4 hydrophobic phenyltriazole moiety of inhibitor **5** is nicely accommodated in an active site groove that is lined with the side-chains of Arg192, Ile210 and Lys254 (Fig. 3a).

Interestingly, in the hPIV-3 HN–**6** complex the reorientation of the Lys254 side-chain towards the 4-methoxymethyltriazole moiety of **6** was observed. This reorientation leads to an additional hydrogen bond formation between the side-chain of Lys254 and the C-4 triazole methoxy oxygen associated with **6**. In addition, the Asn208 side-chain shifts towards the side-chain amino group of Lys254, which establishes a hydrogen bond with the carbonyl oxygen of Asn208 (Fig. 3b). In stark contrast, in the hPIV-3 HN–**5** complex a hydrogen bond between Asp216 and Lys254 was formed and further stabilized the active site (Fig. 3a).

The key catalytic Tyr530 residue is a highly conserved amino acid among all known wild type neuraminidases^20, 21^. The phenolic oxygen of Tyr530 is known to nucleophilically attack the C-2 anomeric carbon of a bound neuraminic acid derivative, as previously described^9^ for the 2,3-difluoroneuraminic acid-based derivative **4**. Accordingly, in the hPIV-3 HN–**5** and hPIV-3 HN–**6** complexes the Tyr530 phenolic oxygen was found at a distance of 2.9 Å and 2.7 Å from the C-2 of inhibitors **5** and **6**, respectively. The structure of the co-crystallized complex with inhibitor **6** had two protein molecules per asymmetric unit (binding site A and B), whereas the structure of the co-crystallized complex with compound **5** had only one protein molecule per asymmetric unit (see Supplementary Methods). Two orientations of Tyr530 were found in binding site A of the hPIV-3 HN–**6** complex: One orientation of the residue Tyr530 was directed towards the binding site (more populated, 52%) and in the second, the residue was oriented away from the binding site. In contrast, in binding site B, Tyr530 was only directed towards the C-2 of inhibitor **6**. No further differences were observed between binding site A and B of the hPIV-3 HN–**6** complex.

Glu549 is one of the seven highly-conserved amino acids in the HN’s active site^21^, and was found oriented away from the binding site in the hPIV-3 HN–**5** complex. In contrast, the Glu549 residue in the hPIV-3 HN–**6** complex was always directed towards the binding site to form a hydrogen bond with an active site-bound water. Interestingly, in the recently described hPIV-3 HN–**4** complex^9^, Glu549 is also found to orient only towards the binding site.

### Effect of the triarginyl cluster shift on inhibitor complexation to the HN protein

The reaction mechanism of the broad NA family has been the subject of intense investigation over the last twenty years^13–15^, especially the IAV NA mechanism^16, 18, 19, 22, 23^. The triarginyl cluster–Neu5Ac carboxylate interaction is thought to have a major influence on the geometry of **1** when bound within the active site^23^. While the Neu2en inhibitors **5** and **6** have planar geometry around C-2 and C-3, as a consequence of their sp^2^-hybridization, 2,3-difluorinated Neu derivatives such as **7** and **8** need to planarise around these carbon atoms upon binding to the enzyme for the catalytic reaction to proceed^19^. As a consequence of the 216-loop movement due to the presence of a C-4 bulky substituent, as seen in the hPIV-3 HN–**5** complex, the induced side-chain rearrangement of Arg 192 results in the loss of a crucial interaction with the carboxyl group of inhibitor **5**. Hence, the introduction of bulky C-4 substituents, such as a phenyltriazole moiety, in 2,3-difluoro Neu substrate-based derivatives (*e.g*. **7**), may in fact be counterproductive and result in poor inhibition of hPIV-3 HN. Conversely, the structure of hPIV-3 HN in complex with inhibitor **6** revealed that the Arg192 side chain remains in close proximity to the inhibitor’s carboxyl group, leading to the expected interactions (Fig. 2b). This structural observation suggests that a 2,3-difluoro derivative of **6** that incorporates a smaller C-4 functionality (*e.g*. **8**), may undergo efficient planarization upon complexation with HN. This planarization should readily promote the expected nucleophilic attack at C-2 of **8** by the phenolic oxygen of Tyr530 to provide the commensurate β-tyrosinyl neuraminide (see Supplementary Figure 2). Finally, this should result in a complex that is similar to what has been previously observed in the recent X-ray crystal structure of the hPIV-3 HN–**4** complex (PDB accession code: 4XJR)^9^ and consequently in a superior antiviral potency, as seen for **4**.

### Synthesis and HN enzyme activity evaluation of the 2,3-difluoro analogues of inhibitors 5 and 6

**Figure 4:**
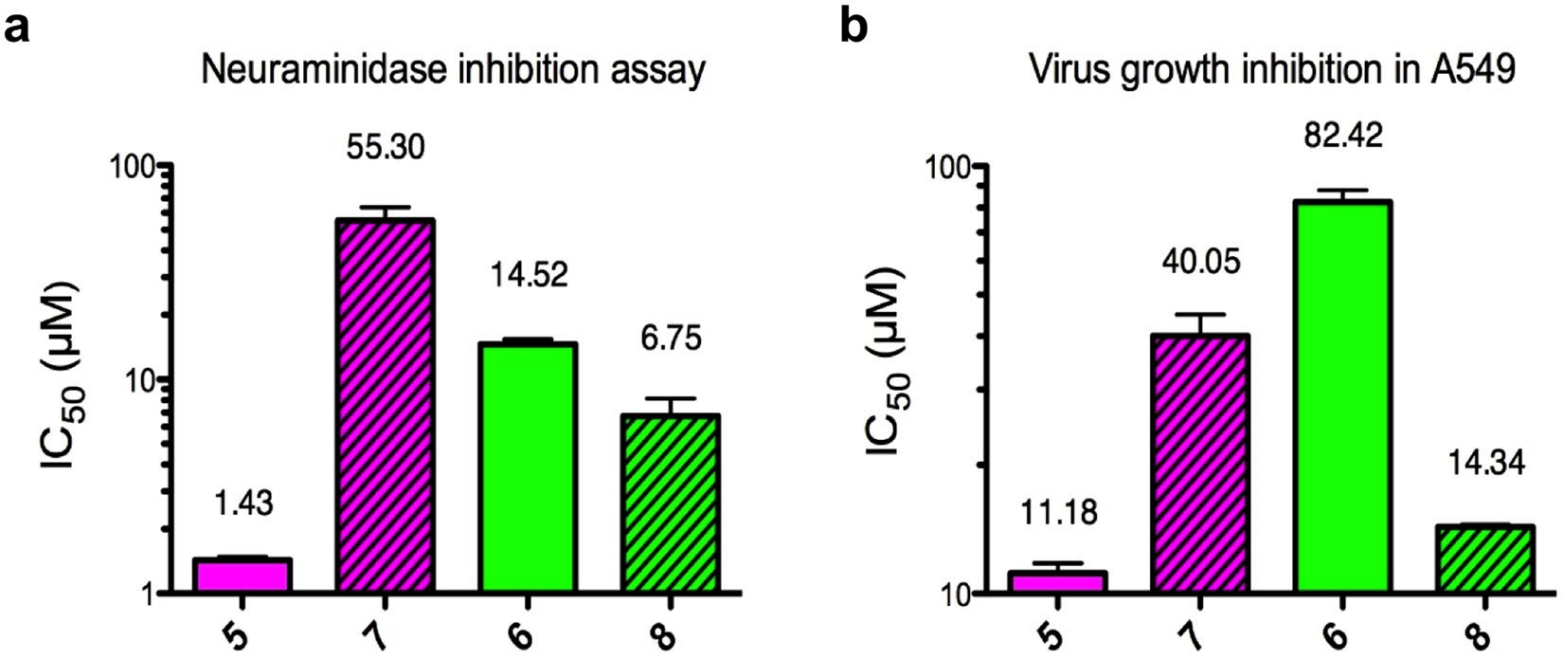
Biological evaluation of the 2,3-difluoro inhibitors in relation to their Neu2en analogues. (**a**) Inhibitors **5**, **6**, **7**, and **8** were evaluated in a neuraminidase assay. These values are the means of determinations from 3 independent experiments performed in duplicates and error bars correspond to calculated standard deviations. (**b**) Virus growth inhibition was determined by an *in situ* ELISA technique using the human cell line A549. IC_50_ values of 11.2 ± 0.9 µM, 40.1 ± 7.0 µM, 82.4 ± 7.7 µM and 14.3 ± 0.3 µM were determined for **5**, **7**, **6** and **8**, respectively. These values were evaluated from at least 2 independent experiments performed in triplicate and error bars correspond to the calculated standard deviation.

To investigate these hypotheses, the 2,3-difluoro analogues **7** and **8** of the potent hPIV-3 inhibitors **5** and **6** were synthesized as shown in Fig. 1b (see Supplementary Methods). Subsequently, neuraminidase inhibition (NI) IC_50_ values were measured by the standard NI fluorescence assay based on the method of Potier *et al*.^24^ using 4-methylumbelliferyl *N*-acetylneuraminide (MUN) as substrate (Fig. 4a)^10^. A 2-fold increase in potency was observed for the novel 2,3-difluoro inhibitor **8** that incorporates the smaller C-4 methoxymethyl triazole substituent (NI IC_50_ = 6.7 µM) compared to its Neu2en analogue (**6**, NI IC_50_ = 14.5 µM). In complete contrast, and as predicted from the analysis of the hPIV-3 HN–**5** structure, a significant loss in potency against hPIV-3 HN neuraminidase function was observed for inhibitor **7** (NI IC_50_ = 55.3 µM compared with an IC_50_ = 1.4 µM for the Neu2en-based inhibitor **5**).

### Inhibition of haemagglutinin function of hPIV-3 HN by 5 and 6 and their 2,3-difluoro derivatives (7 and 8)

Since hPIV-3 HN is a unique multifunctional protein, that possesses both carbohydrate-binding and carbohydrate-processing functionalities in one binding site^25^, we were also interested to explore how difluorination of the inhibitors can affect the hPIV-3 HN haemagglutinin function. For this purpose, virus-mediated agglutination of human red blood cells was evaluated in the presence of decreasing inhibitor concentrations (see Supplementary Methods) in a similar procedure to that previously reported^10^. Results from a haemagglutinin inhibition (HI) assay demonstrated a greatly enhanced potency for the non-fluorinated inhibitors **5** and **6** when compared to their fluorinated analogues **7** and **8** (see Supplementary Figure 3). These significant weaker HI IC_50_ values of the 2,3-difluoro derivatives are in agreement with a recently reported 2,3-difluoro-*N*-acylneuraminic acid^26^ that has a HI IC_50_ value of 1 mM and the biotinylated oligosaccharide, 3′SiaDiLN (Neu5Acα2–3 Galβ1–4GlcNAcβ1–3 Galβ1–4GlcNAc)^27^ that has a K_*d*_ value of 4.3 mM when in complex with soluble hPIV-3 HN. The Neu2en-based inhibitors **5** and **6** are significantly more potent in HI with IC_50_ values of 0.6 µM and 3.9 µM, respectively. Therefore, it seems that planar geometry around C-2 and C-3 of the Neu2en template has an influence on the extent of hPIV-3 HN haemagglutinin function inhibition. Interestingly, the stronger HI for **8** (IC_50_ = 57 µM) when compared to **7** (IC_50_ = 775 µM) at 4 °C may be primarily attributed to the ability of inhibitor **8** to engage the triarginyl cluster and the failure of inhibitor **7** to bind to Arg192, and secondly to the formation of an additional hydrogen bond interaction between the Lys254 side-chain and the C-4 methoxymethyl oxygen of **8**.

### Blockade of hPIV-3 virus propagation in cells by inhibitors 5–8

To further investigate the impact of the C-4 substituent size of the inhibitors on hPIV-3 growth, probes **5**–**8** were evaluated at a cellular level for their capacity to block virus infection and propagation in the human respiratory cell line A549 (lung adenocarcinoma epithelial cells) using our well-established *in situ* ELISA technique^10^. The 2,3-difluoro derivative **8** with the relatively smaller C-4 substituent showed an enhanced capacity to inhibit virus growth, with an experimentally–determined IC_50_ value of 14.3 µM compared to 82.4 µM for the comparable Neu2en inhibitor **6**. This is consistent with the IC_50_ value determinations from NI assay data and is similar to what is observed^9^ for inhibitor **4** with a smaller C-4 azido functionality. Since the C-4 substituents of **4** and **8** are not bulky enough to induce significant movement of the 216-loop, Arg192 remains in its original orientation that maintains the integrity of the triarginyl cluster–Neu carboxylate interactions while maintaining its hydrogen bond to the Cys214 residue (Fig. 2b). In complete agreement with NI enzyme assay data, inferior potency in the virus growth inhibition assay was observed for inhibitor **7** that has the bulkier C-4 phenyltriazole substituent. IC_50_ values of 40.1 µM and 11.2 µM were determined for **7** and its Neu2en analogue **5**, respectively (Fig. 4a).

Finally, all inhibitors were assessed for cytotoxicity to human A549 cells with no toxicity observed at a concentration of 300 µM (data not shown). In addition, we investigated^9^ the capacity of the synthesized inhibitors **5**–**8** to inhibit human neuraminidase 2 (*h*Neu2) compared with the well-described neuraminidase inhibitor Neu5Ac2en (**2**)^28^. No inhibition of *h*Neu2 was observed for either the 2,3-difluoro-*N*-acylneuraminic acid-based inhibitors (**7** & **8**) or their corresponding Neu2en analogues (**5** & **6**) (see Supplementary Figure 4).

## Conclusion

In conclusion, we provide the first structural insights of the hPIV-3 HN protein in a 216-loop open form and describe an in-depth analysis of the rearrangement of binding pocket-resident amino acids as a result of this movement. We have also demonstrated that there is a direct relationship between inhibitor size and the inhibitor’s capacity to open the loop without losing the key interactions between the important active site-based triarginyl cluster and inhibitor. The additional complexity of loop flexibility can directly impact the extent of inhibitor functionalization. In the case of hPIV-3 HN whether large or small C-4 substituents are optimal on the described inhibitors, will depend on the inhibitor framework. From our study “bigger is better” when using the Neu2en framework as the loss of the ionic interaction with Arg192 is compensated by the affinity gain from the hydrophobic C-4 moiety that is oriented in the 216-cavity. In contrast, “smaller is better” when using the 2,3-difluoro-Neu5Ac framework, as these inhibitors are designed to react with the active site Tyr530, thus targeting the catalytic machinery of HN. Smaller substituents at C-4 do not have significant impact on the 216-loop or induce amino acid rearrangement. Consequently, the triarginyl cluster residue Arg192 maintains the capacity to interact with the inhibitor’s carboxylate moiety and facilitates planarization of the substrate-like inhibitor to optimally position C-2 of the difluoro inhibitors for nucleophilic attack by Tyr530.

This study has shown that hPIV-3 HN is a complex flexible protein. The protein’s flexibility, in the context of inhibitor development can be considered a butterfly effect, since a small structural change in one part of the protein has a significant influence on another HN region. This challenge has been recently recognized by the computational biology community^29^. As a consequence, optimal anti-parainfluenza virus drug design that accounts for the hPIV-3 HN protein flexibility and inhibitor-associated structural rearrangements is required. Targeting multiple individual structural features may not lead to the expected synergistic effect, if done without consideration of the impact of structural changes occurring at the whole protein level, and consequently on bound inhibitors. Finally, our findings define an important set of criteria that reveal an critical balancing act with new essential parameters that will assist in the design of novel hPIV-3 HN inhibitors.

## Material and Methods

### General Methods Chemistry

Commercially available reagents and dry solvents were purchased from commercial sources and used without further purification. Anhydrous reactions were carried out using oven-dried glassware and under an argon atmosphere. Reaction progress was monitored through thin layer chromatography (TLC) using aluminium plates pre-coated with Silica Gel 60 F254 (E. Merck). TLC plates were observed under UV light at 254 nm and were then visualized by application of a solution of H_2_SO_4_ in EtOH (5% v/v) followed by heating. Flash chromatography was carried out using Silica Gel 60 (0.040–0.063 mm) and distilled solvents. ^1^H and ^13^C NMR spectra were recorded at 400 and 100 MHz, respectively on a BrukerAvance 400 MHz spectrometer, while ^19^F NMR spectra were recorded at 376 MHz. Chemical shifts (δ) are reported in parts per million, relative to the internal reference residual solvent peak [CDCl_3_: 7.26 (s) for ^1^H, 77.0 (t) for ^13^C; D_2_O: 4.79 (s) for ^1^H]. 2D COSY and HSQC experiments were run when necessary to support assignments. Low-resolution mass spectra (LRMS) were recorded using positive mode, in electrospray ionization mode, on a BrukerDaltonics Esquire 3000 ESI spectrometer. High-resolution mass spectra (HRMS) were recorded for the final derivatives using Griffith University SmartWater Research Centre facility on Agilent 6530 QTOF, with an Agilent 1290 HPLC. Final deprotected derivatives were purified by reversed phase chromatography, using GracePure^TM^ SPE C18-Aq 5000 mg/20 mL and 2% acetonitrile/H_2_O. The purities of all synthetic intermediates after chromatographic purification were > 90% as determined by ^1^H and ^13^C NMR, while the purity of reference compounds synthesized for screening purposes **5** and **6**, and of the new final products **7** and **8** was judged to be ≥ 95%. The synthesis of intermediate **9** (ref. 9) and reference inhibitors **5** and **6** (ref. 10) followed typical literature procedures. The details of synthetic methods used and full characterization data of key intermediates and novel final products are reported here in the supplementary material.

### General procedure for the synthesis of compounds 10 and 11

The 4-azido-4-deoxy-2α,3β-difluoro-5-isobutyramido neuraminic acid intermediate **9** (60 mg, 0.12 mmol) and the corresponding ethynyl derivative (0.24 mmol) were dissolved in a (1:1) mixture of methanol and H_2_O (2 mL). Copper(II)sulfate pentahydrate (6.5 mg, 0.024 mmol) was added to the mixture followed by sodium ascorbate (0.1 mL of freshly prepared 1 M solution in H_2_O). The mixture was heated in a microwave reactor at 80 °C for 30 min. The reaction solvent was then evaporated under vacuum, and the residue was taken up in dichloromethane (50 mL), washed with 10% NH_4_OH (10 mL), followed by brine (10 mL). The organic layer was dried over anhydrous Na_2_SO_4_, concentrated *in vacuo* to give the crude product **10** or **11** and finally purified by silica gel chromatography using a suitable solvent system.

### General procedure for the synthesis of compounds 7 and 8

To compound **10** or **11** (0.068 mmol) in a (1:1) mixture of water and MeOH (2 mL) at 0 °C was added an aq. solution of NaOH (1.0 M) dropwise until a pH of 13–14 was reached. The reaction mixture was allowed to warm up gradually to rt and was stirred at rt overnight. The solution was then rendered acidic with Amberlite^®^ IR-120 (H^+^) resin (to pH = 5), filtered through a cotton plug and washed with MeOH (10 mL) and H_2_O (10 mL). The compound was then purified by reversed phase chromatography, using C18-GracePure^TM^ cartridge and 2% acetonitrile/water, to yield pure **7** (28 mg, 84%) or **8** (21 mg, 68%), as a fluffy white powder after freeze drying.

### Cells and viruses

LLC-MK2 (monkey kidney epithelial cells) cells were maintained at 37 °C, 5% CO_2_ in EMEM (Lonza, Basel, Switzerland) supplemented with 1% Glutamine (1% final concentration) and 2% heat-inactivated fetal bovine serum (FBS). A549 cells (human lung adenocarcinoma epithelial cells) were provided by the European Collection of Cell Cultures (86012804–1VL, Sigma-Aldrich). Cells were propagated in Dulbecco’s Modified Eagle Medium (DMEM) (Lonza, Basel, Switzerland) supplemented with of 1% Glutamine and 5% FBS. For infection procedures, A549 cells were maintained in DMEM supplemented with 1% Glutamine only.

hPIV-3 (strain C-243) was purchased from the American Type Culture Collection (Manassas, VA) and virus stock was amplified in LLC-MK2 cells at 35 °C, 5% CO_2_ in EMEM (only supplemented 1% Glutamine). After 96 hours of incubation, the virus-containing supernatant was harvested, clarified and purified by PEG precipitation followed by sucrose gradient fractionation as previously published^10^.

### hPIV-3 HN inhibitors

Compounds **2**–**8** were each provided as a lyophilized powder and then reconstituted in sterile water to generate a 10 mM stock solution. Solutions were sonicated for 15 min to allow complete dissolution and then filter-sterilized. The stock solution was stored in a glass vial at −20 °C and freshly diluted in appropriate buffer before usage.

### Recombinant human Neuraminidase 2 (*h*Neu2) expression and purification

The entire coding sequence of *h*Neu2 was cloned into pGEX-2T expression vector (Amersham Biosciences). Recombinant *h*Neu2 expression was performed in *E. Coli* cells. Purification of recombinant *h*Neu2 was accomplished in three steps using affinity chromatography, followed by two successive steps of high resolution anion and cation exchange chromatography as previously described^12, 30^.

### Neuraminidase inhibition assay

Viral neuraminidase inhibition (NI) assays were accomplished with purified hPIV-3 in reaction buffer/hPIV3 (NaOAc 50 mM, CaCl_2_ 5 mM, pH 4.6). The maximal fluorescence signal of purified virus (positive control) was required to be at least 5 times higher than the fluorescence background (negative control) in order to be considered as statistically relevant. NI assays were performed in triplicate following the protocol as described earlier^9, 10^.

The NI assay with human Neuraminidase 2 (*h*Neu2) was performed as previously described^9^, using 60 ng of purified *h*Neu2 enzyme in reaction buffer/*h*Neu2 (NaOAc 50 mM, pH 5.5), supplemented with 10 μg BSA and 0.5 mM MUN per reaction. All inhibitors were tested in duplicate in the concentration range from 0.001 μM to 1000 μM. After 10 min of incubation with agitation (1000 rpm) at 37 °C, the enzymatic reaction was stopped with glycine buffer (glycine 0.25 M, pH 10.4) and the product fluorescence was measured in relative fluorescence units (RFU) using a Victor 3 multilabel reader (PerkinElmer, Waltham, MA). Controls were conducted with MUN and purified *h*Neu2, the enzymatic reaction was stopped at t = 0 for the negative control and at t = 10 min for the positive control.

The obtained data were normalised by background (negative control RFU) subtraction and processed data were analyzed with GraphPadPrism 4 (GraphPad Software Inc., La Jolla, CA) to calculate IC_50_ values. The concentration of inhibitor that reduced the neuraminidase activity by 50% compared to those of a non-treated virus/protein suspension was considered as the NI IC_50_ value.

### Haemagglutination inhibition assay

The HN inhibitors were assessed in haemagglutination inhibition assays using human red blood cells (RBC) as previously described^10^ with minor modifications. The lowest final concentration of hPIV-3 that leads to a complete agglutination of human-RBC at 4 °C is considered as 1 HAU. 4 haemagglutination units (HAU) of hPIV-3 (final 1 HAU per well) were pre-mixed with inhibitors of each tested concentration (4X dilution in PBS) on ice. Next, an equivalent volume of ice-cold 1% human-RBC in PBS was added to each well of the inhibitor/virus pre-mixture and the plate was then incubated for 90 min at 4 °C before determining the extent of haemagglutination inhibition. The observed agglutination in a control well containing only 0.5 HAU of hPIV-3 and human-RBC was used to determine the concentration of the inhibitor that reduced the haemagglutination activity (agglutination) by 50% compared to those of a non-treated virus suspension. A similar observed agglutination as in the control well (0.5 HAU of hPIV-3) was considered as the HI IC_50_ value for each respective inhibitor.

### *In situ* ELISA

This technique was extensively used in previous studies^9, 10^, to assess dose-dependent potency of compounds to inhibit virus growth by measuring expression levels of hPIV-3 HN at the cell surface of an infected cell monolayer. This method is shortly summarized here; infection was performed with 100 FFU/well of hPIV-3 for 1 h at 37 °C with gentle agitation every 15 min on a confluent A549 monolayer seeded in a 96 well-plate. Assays were performed in triplicate; inocula were removed and replaced with respective compound dilutions (final concentration of 0.001 μM to 1000 μM). Virus proliferation was allowed at 37 °C, 5% CO_2_ for 36–40 h. Virus was inactivated and cells were fixed by treatment with 4% paraformaldehyde in PBS for 20 min. Next, 0.3% H_2_O_2_/PBS (final concentration) was added to inactivate endogenous peroxidases at 37 °C for 30 min. 1:2000 dilution of mouse monoclonal IgG anti-hPIV-3HN (Fitzgerald, clone# M02122321, 2.0 mg/mL) in 5% milk/PBS was incubated with the cells for 1 h at 37 °C, followed by an incubation of 1:2000 dilution of goat anti- Mouse-IgG(H + L)-HRP conjugate (BioRad, ref# 170–6516) in 5% milk/PBS for 1 h at 37 °C. BD OptEIATMB substrate (BD Biosciences, San Jose, CA, 100 μL) was added to virus-infected cells and the reaction was stopped after 3–5 min by addition of 1 M H_2_SO_4_ (50 μL). The initial absorbance (OD) of each well was read at 450 nm for 0.1 s using a Victor 3 multilabel reader. The negative control (non-infected cells) OD was subtracted from the initial ODs and final OD values were analyzed with GraphPadPrism4 to calculate IC_50_ values. The IC_50_ value was considered as the concentration of inhibitor that reduced the absorbance at 450 nm by 50% compared to a non-treated infected cell monolayer.

### Cytotoxicity assay

The cytotoxcity of all compounds, described in cell-based infection assays, was assessed by incubating the inhibitors at two different concentrations (30 µM and 300 µM) with the human cell line A549 for 48 h. The cell viability was measured by adding AlamarBlue® (life technologies) reagent as 10% of the sample volume to the cells and incubate them for 4 h at 37 °C, following fluorescence was read at 570 nm with a BioRad plate reader.

### Recombinant HN expression and purification

The ectodomain of the haemagglutinin-neuraminidase protein from human parainfluenza virus type 3 Washington/1957 C243 was expressed using the Bac-to-Bac^®^ baculovirus expression system (Invitrogen, Carlsbad, CA). The nucleotide sequence for a honeybee melittin signal peptide (HBM) was added downstream to the sequence encoding for the HN ectodomain (amino acids 125 to 572). This sequence (HBM + HN) was codon optimised for expression in *Spodoptera frugiperda* cells (Sf9) and ordered directly through the DNA2.0 gene synthesis service (DNA2.0, Menlo Park, CA) as a gene named HBM-HNhPIV-3opt. HBM-HNhPIV-3opt was amplified by PCR and ligated into a pFastBac⁄CT-TOPO® vector that provides an additional C-terminal 6-histidine tag (His-Tag) for purification and detection purposes. The generation and amplification of recombinant baculovirus containing HBM-HNhPIV-3opt were performed according to the manufacturer’s instructions. Sf9 cells (Invitrogen), cultured in Insect-XPRESS protein free insect cell medium (Lonza), were infected with high MOI of HBM-HNhPIV-3opt baculovirus. Four days post-infection the supernatant, containing recombinant HN, was collected to yield the highest protein expression. The supernatant was clarified by centrifugation (3000 RCF for 15 min) to remove cell debris and then purified on a HisTrap excel 5 mL column (GE Healthcare life sciences, Buckinghamshire, England) following the manufacturer’s protocol. Recombinant HN was eluted with 500 mM imidazole solution and collected fractions were assessed by a neuraminidase activity (NA) assay (see below). The most active fractions were pooled and concentrated with a 10 kDa Amicon Ultra filter unit (Millipore) to a final volume of 800 μL. An additional purification step was performed that employed fast protein liquid chromatography (Amersham Biosciences) over a Superdex 75 gel filtration column (GE Healthcare) at 4  °C and 1 mL fractions were collected with a Frac-920. Protein-containing fractions, as determined by monitoring fraction collection at 280 nm, were assessed in a NA assay as well as subjected to SDS-PAGE. Purified and concentrated recombinant HN protein was stored at 4 °C.

### Crystallization of hPIV-3 HN co-complexes

The 4 mg/mL hPIV-3 HN protein stock solution was pre-incubated with a final concentration of 1 mM inhibitor **5** or 1.5 mM inhibitor **6** in 0.1 M citrate buffer pH 4.6, 0.2 M ammonium sulfate and 15% PEG 3000. Crystallization trials were set up as 2 µL pre-incubated stock solution using the hanging drop vapour diffusion method. The drop was equilibrated against a 500 µL reservoir (0.1 M citrate buffer pH 4.6, 0.2 M ammonium sulfate and 15% PEG 3000). The crystals were mounted in nylon loops (Hampton Research) and flash frozen at 100 K in a cryoprotectant solution containing 20% glycerol in addition to the precipitant solution.

### Data collection and structure determination

X-ray diffraction data were collected at the Photon Factory AR-NW12A^31^ and BL5A beam lines (Japan) at a wavelength of 1.000 Å. Images for the complex hPIV-3 HN-**5** were recorded on an ADSC Q315r CCD detector using 5 s exposure per 0.5° oscillation angles. In case of hPIV-3 HN-**6**, images were recorded on an ADSC Q210r CCD detector using a 5 s exposure per 1° oscillation angles. The data were reduced and scaled using XDS^32^ and CCP4 (ref. 33) program suites. The hPIV-3 HN co-complexes were determined by molecular replacement using Phaser^34^ from the Phenix suite^35^ employing the atomic coordinates of hPIV-3 HN (PDB ID: 4XJQ) as the search model. Coot^36^, Refmac5^37^ and Phenix.refine^35^ were employed for initial restrained, model-building and further cycles of refinement. The final models were confirmed and validated through the Molprobity^38^ server. X-ray data collection statistics are summarized in Supplementary Table 1. Atomic coordinates and structure factors have been deposited in the Protein Data Bank (PDB) under the accession codes 5KV8 and 5KV9 for the hPIV-3 HN–**5** and hPIV-3 HN–**6** complex structures, respectively.

Structures of co-crystallized complexes with inhibitor **6** belonged to the *P*212121 space-group with unit cell dimensions of 81.57 Å × 98.61 Å × 103.39 Å and had two protein molecules per asymmetric unit as previously described for the *apo*-hPIV-3 HN structure (PDB accession﻿ code: 4XJQ)^9^ and for all reported hPIV-3 HN complexes to date^9, 21, 26, 39^. In contrast, for the first time, structures of co-crystallized complexes with compound **5** were found to belong to the *C*2221 space-group with unit cell dimensions of 80.10 Å × 107.37 Å × 94.49 Å and one protein molecule per asymmetric unit was observed. The structure of the *C*2221 crystal form was determined by the molecular replacement method using a monomer of apo-hPIV3-HN (PDB accession code: 4XJQ) as a search model. Analysis of the *P*212121 and *C*2221 crystal forms revealed that the overall packing of molecules are the same, but ligand induced conformational differences in the active site region of hPIV3-HN lead to a change in the symmetry axis between the molecules in the protein biological dimer from non-crystallographic in the *apo* form to crystallographic in the ligand-bound form resulting in a *C*2221 space-group.

## Electronic supplementary material

Supplementary Information
