# Supplementary material for: The impact of the butterfly effect on human parainfluenza virus haemagglutinin-neuraminidase inhibitor design
Source: Sci Rep. 2017 Jul 3;7:4507. doi: 10.1038/s41598-017-04656-y (PMC5495814; doi:10.1038/s41598-017-04656-y)
Supplement: Supplementary file 1 — Supplementary Information [file 41598_2017_4656_MOESM1_ESM.pdf]

The impact of the butterfly effect on human parainfluenza virus haemagglutinin-neuraminidase inhibitor design.

Larissa Dirr[1], Ibrahim M. El-Deeb[1], Leonard M. G. Chavas[2], Patrice Guillon[1] and Mark von Itzstein[1].*

[1]*Institute for Glycomics, Griffith University, Gold Coast Campus, Queensland, 4222, Australia.*

[2]*Experiments division, Synchrotron SOLEIL, Gif-sur-Yvette, France.*

SUPPLEMENTARY INFORMATION

SUPPLIMENTARY SCHEMES AND FIGURES

Synthesis of Compounds 7 and 8

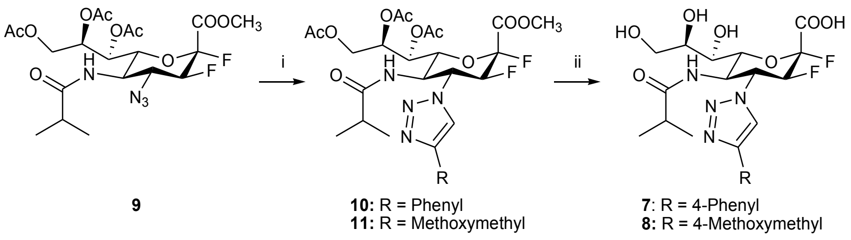

Supplementary Figure 1. *Reagents and Conditions:* (i) $CuSO_4$, sodium ascorbate, MeOH/H_2O (1:1), M.W., 80 °C, 30 min (**10**, 89%; **11**, 81%); (ii) NaOH, MeOH/H_2O (1:1), rt, o/n (**7**, 84%; **8**, 68%).

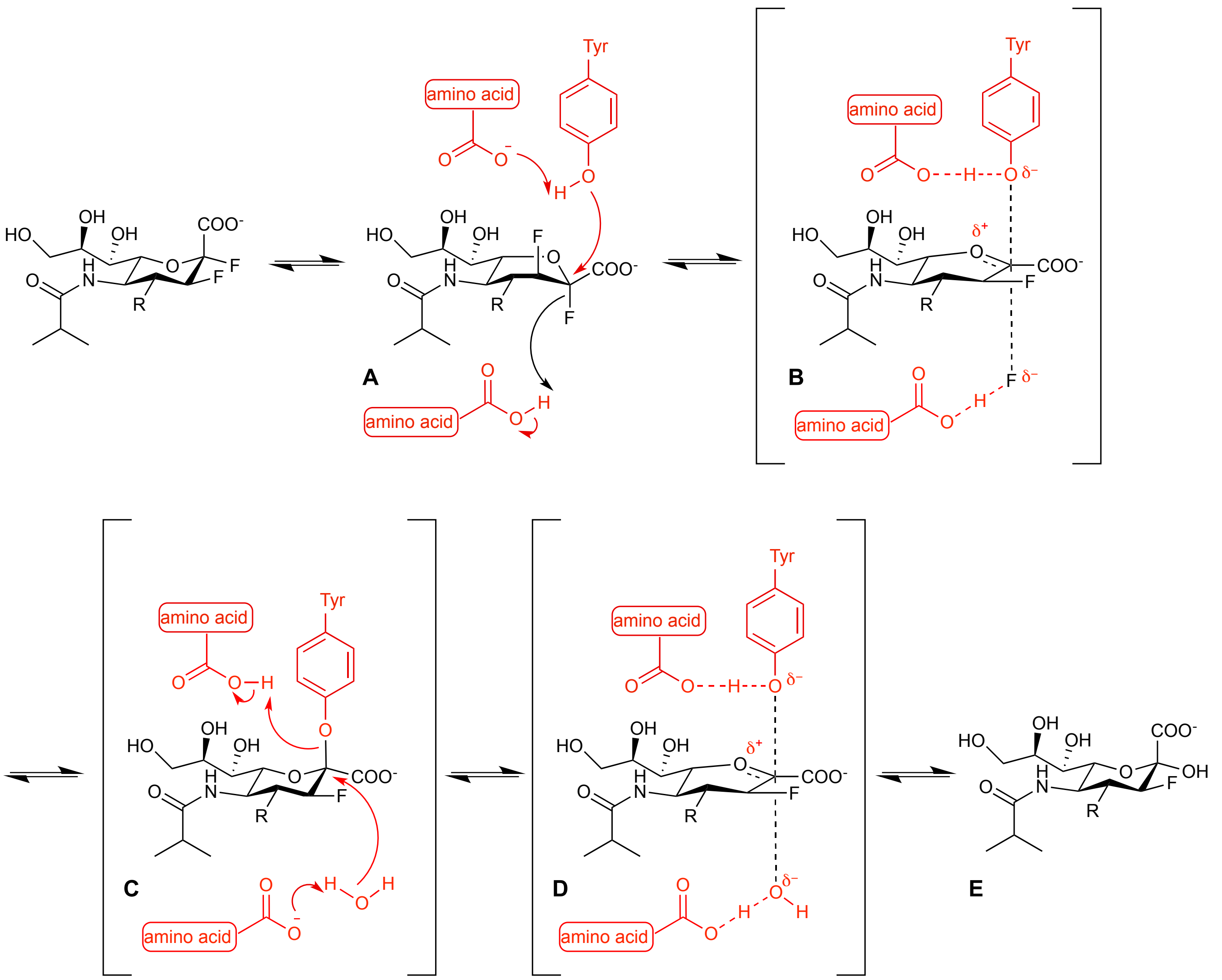

Supplementary Figure 2: A general catalytic hydrolysis mechanism of human parainfluenza virus haemagglutinin-neuraminidase for the 2,3-difluoro-Neu5Acyl substrates.

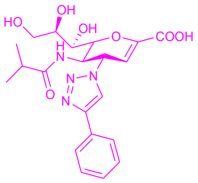 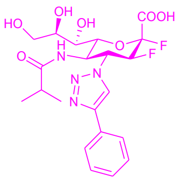 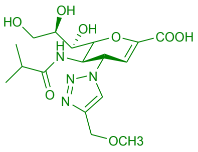 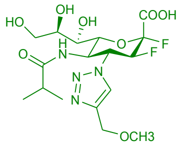

IC_{50} values (μM)

	**5**	**7**	**6**	**8**
HI	0.63 ± 0.17	775 ± 35.3	3.88 ± 0.18	57 ± 9.9

Supplementary Figure 3. Haemagglutination inhibition (HI) assay. Comparison of HI IC_{50} values of compounds **5** - **8**. Greater potency is observed for Neu2en compounds **5** and **6** compared to their corresponding 2,3-difluoro analogues **7** and **8**.

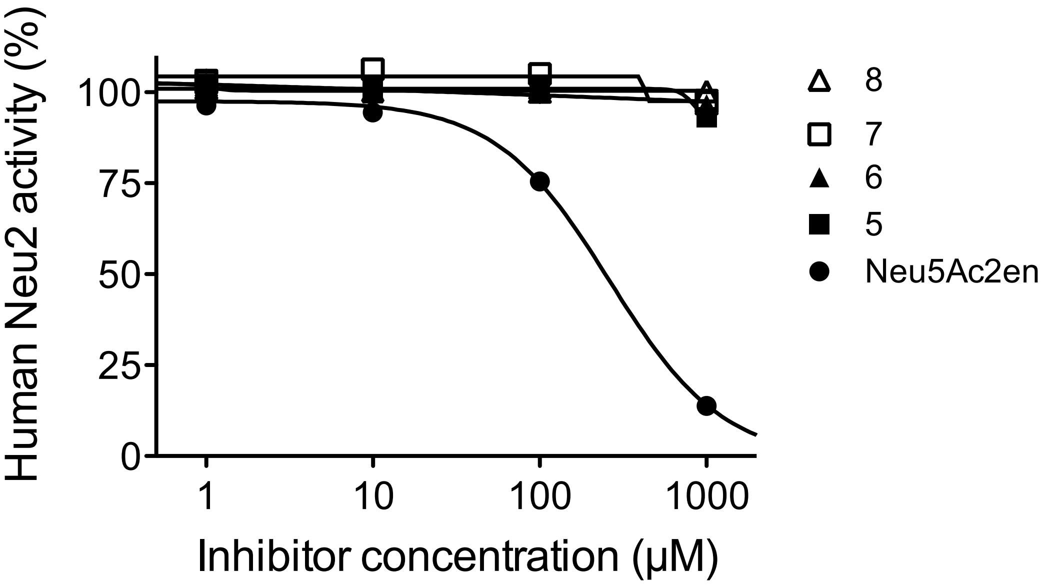

Supplementary Figure 4. Human Neuraminidase 2 (Neu2) inhibition. Activity of the human neuraminidase 2 was tested in presence of inhibitors **5 - 8** and the reference compound Neu5Ac2en (**2**).

Methyl 7,8,9-tri-*O*-acetyl-3,4,5-trideoxy-3-fluoro-5-isobutyramido-4-(4-phenyl-[1,2,3,]triazol-1-yl)-D-*erythro*-β-L-*gluco*-non-2-ulopyranosylonate fluoride (10).

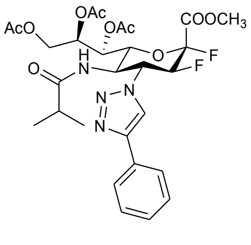

10

Purification by silica gel chromatography using hexane/acetone (2:1) yielded (66 mg, 89%) of pure **10.** ^{1}H NMR (400 MHz, $CDCl_3$): δ 0.95 (d, *J* = 6.8 Hz, 3H, isobut-CH_3), 1.01 (d, *J* = 6.9 Hz, 3H, isobut-CH_3), 2.02 (s, 3H, OAc), 2.11 (s, 3H, OAc), 2.14–2.27 (m, 4H, isobut-CH, OAc), 3.88–3.96 (s, 4H, $COOCH_3$, H-5), 4.20–4.31 (m, 2H, H-9, H-9’), 5.05–5.32 (m, 3H, H-3, H-6, H-7), 5.40 (ddd, *J* = 8.9, 4.3, 2.4 Hz, 1H, H-8), 5.92 (d, *J* = 7.3 Hz, 1H, NH), 6.37 (q, *J* = 11.2 Hz, 1H, H-4), 7.35 (dd, *J* = 8.4, 6.2 Hz, 1H, Ph-H), 7.43 (t, *J* = 7.5 Hz, 2H, 2Ph-H), 7.69–7.90 (m, 3H, 2Ph-H, triazole-CH); ^{13}C NMR (100 MHz, $CDCl_3$): δ 18.94 (isobut-CH_3), 19.31 (isobut-CH_3), 20.59, 20.74, 20.87 (3 OCO$\underline{CH_3}$), 35.68 (isobut-CH), 51.24 (dd, *J* = 6.1, 2.0 Hz, C-5), 53.62 ($COOCH_3$), 58.74 (dd, *J* = 19.1, 8.3 Hz, C-4), 61.83 (C-9), 67.19 (C-7), 68.12 (C-8), 71.24 (d, *J* = 2.1 Hz, C-6), 90.77 (dd, *J* = 196.1, 30.0 Hz, C-3), 105.63 (dd, *J* = 230.5, 26.8 Hz, C-2), 121.75 (triazole-CH), 125.74 (Ph), 128.51 (Ph), 128.94 (Ph), 129.92 (Ph q C), 147.64 (triazole-q C), 164.54 (d, *J* = 32.9 Hz, $\underline{CO}OCH_3$), 169.49, 170.39, 171.00 (3 O$\underline{CO}CH_3$), 178.42 (isobut-CO); LRMS [$C_{28}H_{34}F_2N_4O_{10}$] (*m/z*): (+ve ion mode) 647.4 $[M+Na]^+$.

Methyl 7,8,9-tri-*O*-acetyl-3,4,5-trideoxy-3-fluoro-5-isobutyramido-4-(4-methoxymethyl-[1,2,3,]triazol-1-yl)-D-*erythro*-β-L-*gluco*-non-2-ulopyranosylonate fluoride (11).

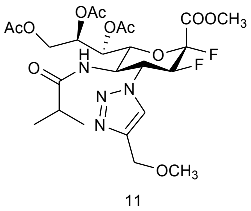

11

Purification by silica gel chromatography using hexane/acetone (3:2) yielded (57 mg, 81%) of pure **11**. ^{1}H NMR (400 MHz, $CDCl_3$): δ 0.96–1.01 (m, 6H, isobut-2CH_3), 2.01 (s, 3H, OAc), 2.09 (s, 3H, OAc), 2.13 (s, 3H, OAc), 2.23 (dq, *J* = 13.0, 6.8 Hz, 1H, isobut-CH), 3.36 (s, 3H, OCH_3), 3.90–3.96 (m, 4H, $COOCH_3$, H-5), 4.19 (dd, *J* = 12.6, 4.6 Hz, 1H, H-9), 4.27 (dd, *J* = 12.6, 2.5 Hz, 1H, H-9’), 4.54 (s, 2H, OCH_2), 4.99–5.30 (m, 3H, H-3, H-6, H-7), 5.37 (ddd, *J* = 8.6, 4.6, 2.4 Hz, 1H, H-8), 6.19–6.29 (m, 2H, H-4, NH), 7.60 (s, 1H, triazole-CH); ^{13}C NMR (100 MHz, $CDCl_3$): δ 18.86 (isobut-CH_3), 19.32 (isobut-CH_3), 20.59, 20.75, 20.79 (3 OCO$\underline{CH_3}$), 35.53 (isobut-CH), 50.84 (d, *J* = 5.8 Hz, C-5), 53.62 (COO$\underline{CH_3}$), 58.34 (OCH_3), 59.00–59.40 (m, C-4), 61.86 (C-9), 65.50 (OCH_2), 67.08 (C-7), 68.29 (C-8), 71.51 (C-6), 90.64 (dd, *J* = 196.1,

30.3 Hz, C-3), 105.58 (dd, J = 230.3, 26.8 Hz, C-2), 124.52 (triazole-CH), 144.83 (triazole-q carbon), 164.51 (d, J = 32.9 Hz, <u>CO</u>OCH$_3$), 169.56 , 170.41, 170.69 (3 O<u>CO</u>CH$_3$), 178.35 (isobut-CO); ^{19}F NMR (376 MHz, CDCl$_3$): δ -118.67 (d, J = 14.0 Hz, F-2α), -197.42 (d, J = 13.2 Hz, F-3β); LRMS [$C_{24}H_{34}F_2N_4O_{11}$] (m/z): (+ve ion mode) 615.3 [M+Na]$^+$.

3,4,5-Trideoxy-3-fluoro-5-isobutyramido-4-(4-phenyl-[1,2,3,]triazol-1-yl)-D-*erythro*-β-L-*gluco*-non-2-ulopyranosonic fluoride (7).

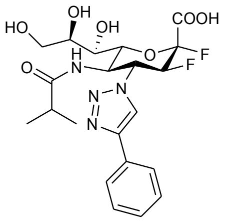

7

^{1}H NMR (400 MHz, CD$_3$OD): δ 0.77 (d, J = 6.9 Hz, 3H, isobut-CH$_3$), 0.95 (d, J = 6.8 Hz, 3H, isobut-CH$_3$), 2.28 (dt, J = 13.7, 7.0 Hz, 1H, isobut-CH), 3.48 (d, J = 9.1 Hz, 1H, H-7), 3.61 (dd, J = 11.6, 5.8 Hz, 1H, H-9), 3.74–3.84 (m, 2H, H-8, H-9'), 4.58 (d, J = 10.4 Hz, 1H, H-6), 4.85 (m, 1H, H-5), 5.15 (ddd, J = 50.7, 13.7, 9.8 Hz, 1H, H-3), 5.91 (q, J = 10.9 Hz, 1H, H-4), 7.34 (t, J = 7.4 Hz, 1H, Ph-H), 7.43 (t, J = 7.6 Hz, 2H, 2Ph-H), 7.81 (d, J = 7.7 Hz, 2H, 2Ph-H), 8.50 (s, 1H, triazole-CH); ^{13}C NMR (100 MHz, CD$_3$OD): δ ^{13}C NMR (101 MHz, MeOD) δ 17.89 (isobut-CH$_3$), 18.51 (isobut-CH$_3$), 34.74 (isobut-CH), 48.83 (d, J = 6.0 Hz, C-5), 63.40 (dd, J = 18.0, 8.5 Hz, C-4), 63.71 (C-9), 68.65 (C-7), 70.01 (C-8), 74.10 (d, J = 2.9 Hz, C-6), 90.93 (dd, J = 193.0, 31.4 Hz, C-3), 106.79 (dd, J = 211.6, 28.4 Hz, C-2), 120.83 (triazole-CH), 125.30 (Ph), 127.99 (Ph), 128.55 (Ph), 130.19 (Ph q C), 147.42 (triazole-q C), 168.40 (d, J = 29.0 Hz, COOH), 179.40 (isobut-CO); ^{19}F NMR (376 MHz, CD$_3$OD): δ -115.20 (d, J 14.6 Hz, F-2α), -200.90 (d, J = 14.6 Hz F-3β); LRMS [$C_{21}H_{25}F_2N_4NaO_7$] (m/z): (+ve ion mode) 529.2 [M+Na]$^+$; HRMS (API) (m/z): [M+1]$^+$ calcd for $C_{21}H_{26}F_2N_4O_7$ [M+1]$^+$ 483.1697; found, 483.1721.

3,4,5-Trideoxy-3-fluoro-5-isobutyramido-4-(4-methoxymethyl-[1,2,3,]triazol-1-yl)-D-*erythro*-β-L-*gluco*-non-2-ulopyranosonic fluoride (8).

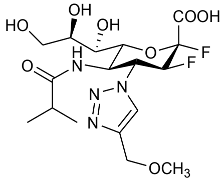

8

^{1}H NMR (400 MHz, D$_2$O): δ 0.81 (d, J = 6.9 Hz, 3H, isobut-CH$_3$), 0.94 (d, J = 6.9 Hz, 3H, isobut-CH$_3$), 2.35 (p, J = 6.9 Hz, 1H, isobut-CH), 3.38 (s, 3H, OCH$_3$), 3.54 (d, J = 9.2 Hz, 1H, H-7), 3.61 (m, 1H, H-9), 3.78–3.91 (m, 2H, H-8, H-9'), 4.52–4.76 (m, 3H, OCH$_2$, H-6), 4.85 (m, 1H, H-5), 5.32 (ddd, J = 49.5, 13.7, 9.7 Hz, 1H, H-3), 5.75 (q, J = 11.2 Hz, 1H, H-4), 8.28 (s, 1H, triazole-CH); ^{13}C NMR (100 MHz, D$_2$O): δ

18.11 (isobut-CH_3), 18.65 (isobut-CH_3), 34.87 (isobut-CH), 48.15 (d, J = 6.1 Hz, C-5), 57.26 (OCH_3), 63.16 (C-9), 63.18–63.58 (m, C-4), 64.13 (OCH_2), 67.85 (C-7), 69.83 (C-8), 73.73 (d, J = 3.3 Hz, C-6), 90.22 (dd, J = 190.9, 32.6 Hz, C-3), 106.79 (dd, J = 224.1, 27.8 Hz, C-2), 124.89 (triazole-CH), 144.09 (trizaole-q C), 169.21 (d, J = 30.7 Hz, COOH), 180.88 (isobut-CO); ^{19}F NMR (376 MHz, D_2O): δ -112.75 (d, J = 13.8 Hz, F-2α), -199.41 (d, J = 14.3 Hz, F-3β); LRMS [$C_{17}H_{25}F_2N_4NaO_8$] (*m/z*): (+ve ion mode) 496.8 $[M+Na]^+$; HRMS (API) (*m/z*): $[M+1]^+$ calcd for $C_{17}H_{26}F_2N_4O_8$ $[M+1]^+$ 453.1791; found, 453.1810

X-ray data collection and refinement statistics

Supplementary Table 1. X-ray data collection and refinement statistics for the hPIV-3 HN–**5** and hPIV-3 HN–**6** complexes. Data in brackets correspond to the highest resolution shells.

	**hPIV-3 HN–5**	**hPIV-3 HN–6**
**Data collection**		
Beam line	BL5A	AR-NW12A
Wavelength (Å)	1.000	1.000
Space group	*C*2221	*P*212121
Cell parameters<br>a, b, c (Å)<br>α, β, γ (º)	 80.10, 107.37, 94.49<br>90, 90, 90	 81.57, 98.61,103.39<br>90, 90, 90
Resolution (Å)	36.87 -2.00	45.78-1.95
Observed reflections	98,693	117,575
Unique reflections	24,135	61,585
Completeness (%)	86.6 (78.0)	99.9 (99.1)
Average I/σ(I)	17.8 (5.2)	16.5 (2.0)
Rmerge (%)	6.1 (25.9)	9.9 (79.3)
Rmeas (%)	7.0 (30.3)	10.7 (87.5)
Rpim (%)	3.3 (15.2)	4.0 (36.2)
CC1/2	0.998 (0.907)	0.999 (0.710)
CC*	0.999 (0.973)	1.000 (0.889)
**Refinement**		
R-work	0.163 (0.180)	0.159 (0.268)
R-free	0.205 (0.259)	0.198 (0.312)
RMS bond (Å)	0.009	0.009
RMS angle (º)	1.286	1.223
Ramachandran favoured (%)	94.8	95.28
Ramachandran outliers (%)	0.7	0.0
Average B-factor (Å ^{2})<br>Macromolecules<br>Ligands<br>I57<br>I40 site A<br>I40 site B<br>Solvent	 22.0<br><br>23.01<br><br><br>26.02	 26.54<br><br><br>30.40<br>34.28<br>33.51
Wilson B-factor (Å ^{2})	18.7	23.9
Solvent content (%)	47.0	47.0
Water Molecules	252	643
PDB entry	5KV8	5KV9

^{1}H and ^{13}C NMR spectra of new intermediates and final products

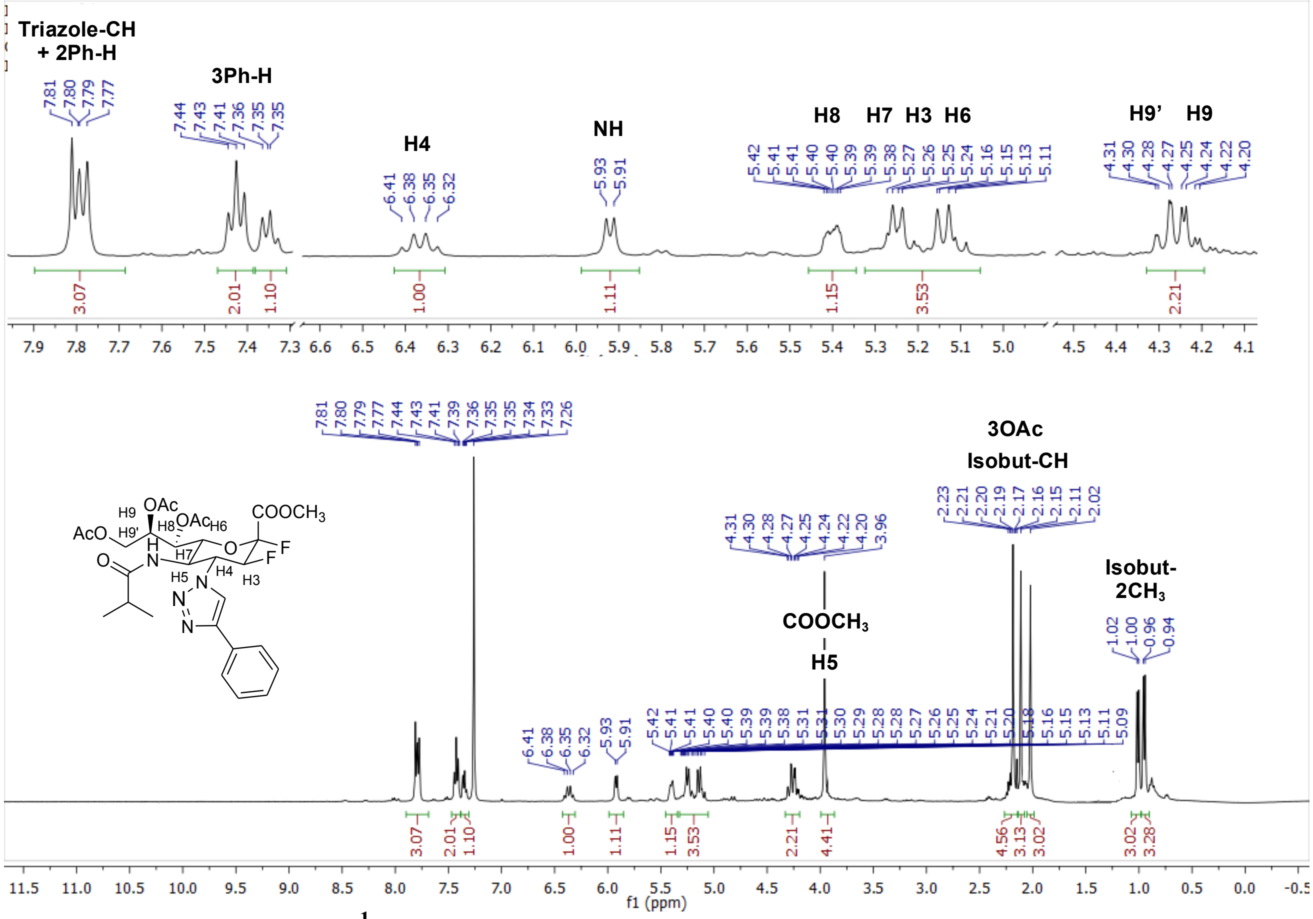

^{1}H NMR spectrum of 10 (400 MHz, $CDCl_3$)

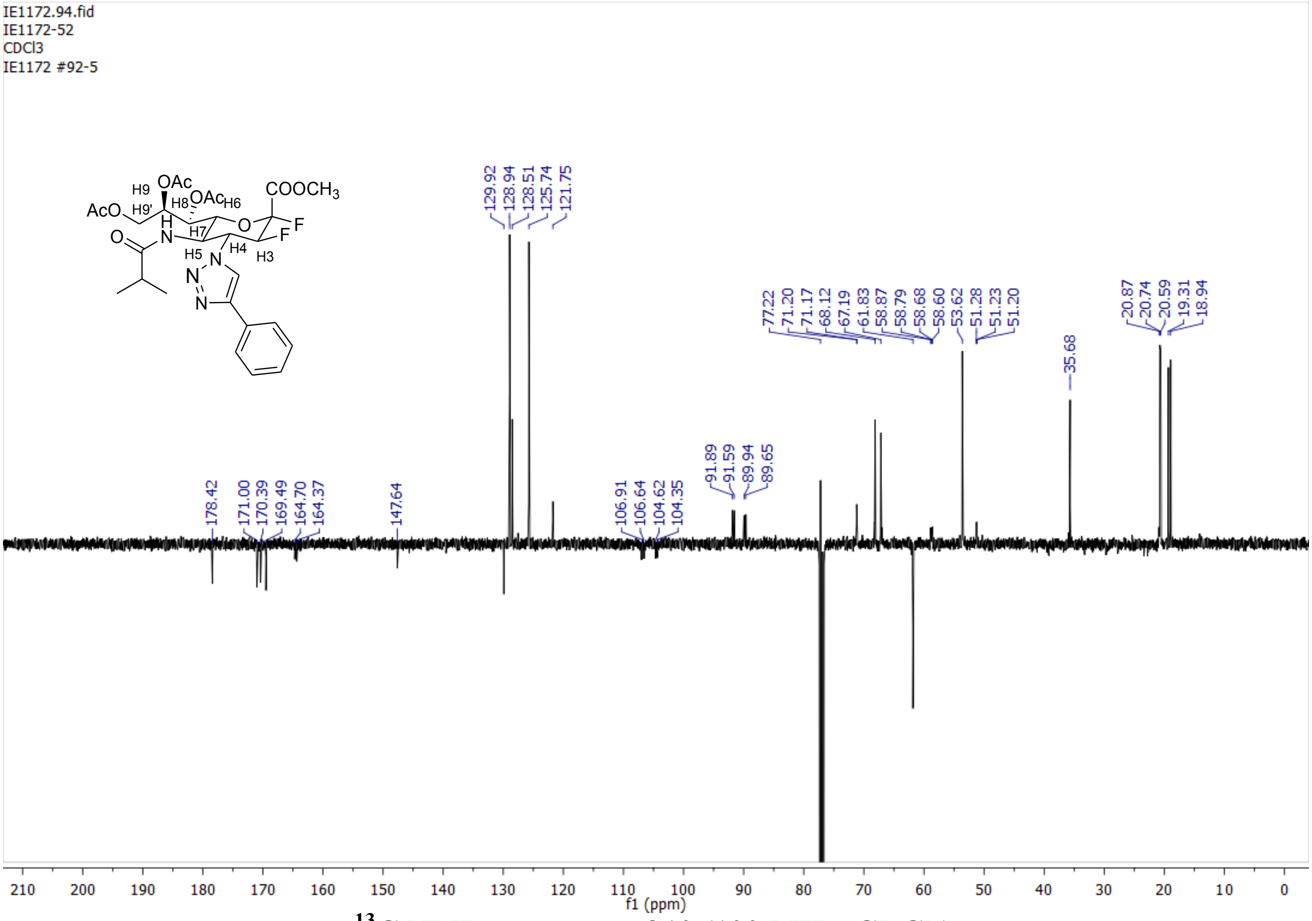

^{13}C NMR spectrum of 10 (100 MHz, $CDCl_3$)

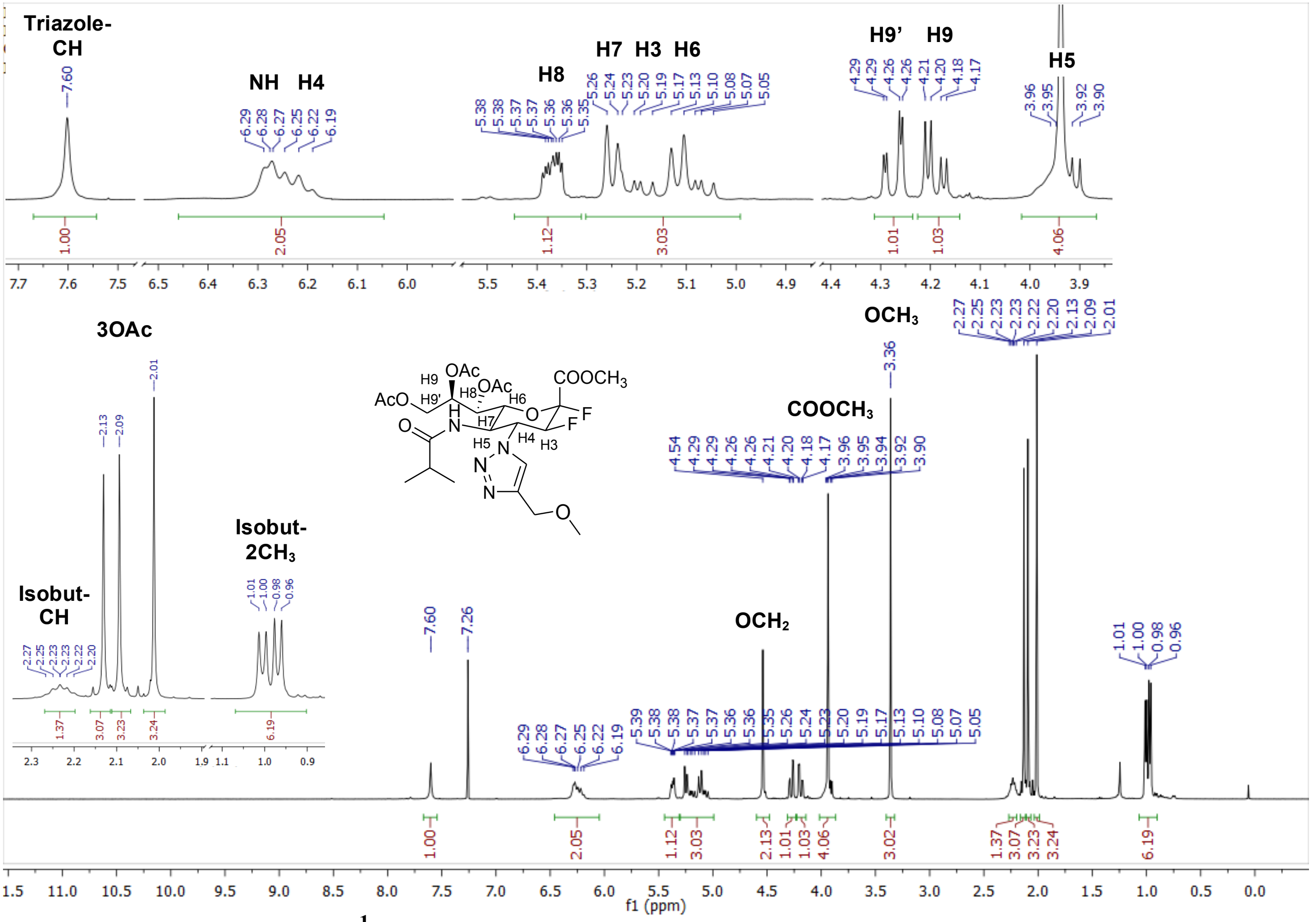

^{1}H NMR spectrum of 11 (400 MHz, $CDCl_3$)

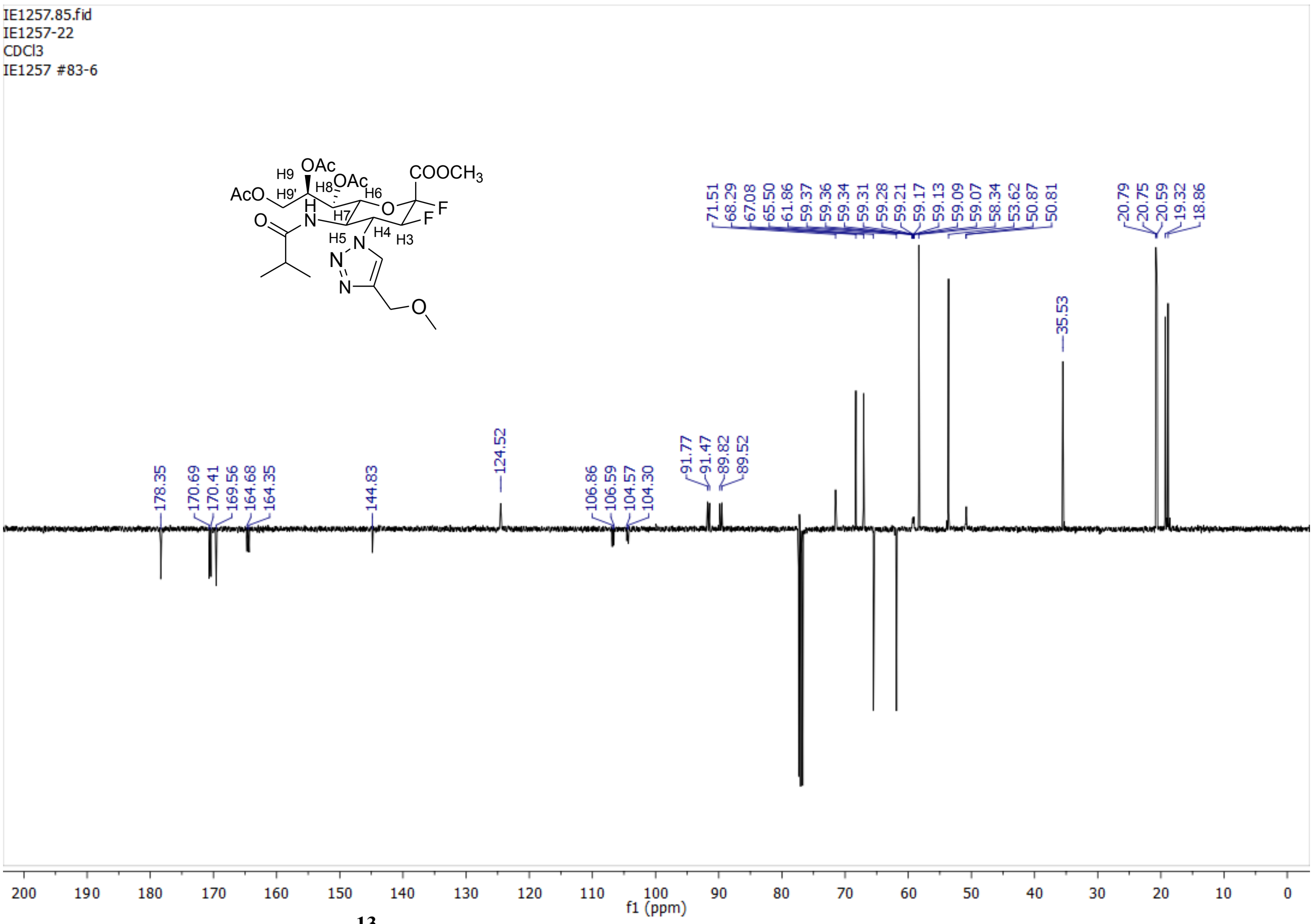

^{13}C NMR spectrum of 11 (100 MHz, $CDCl_3$)

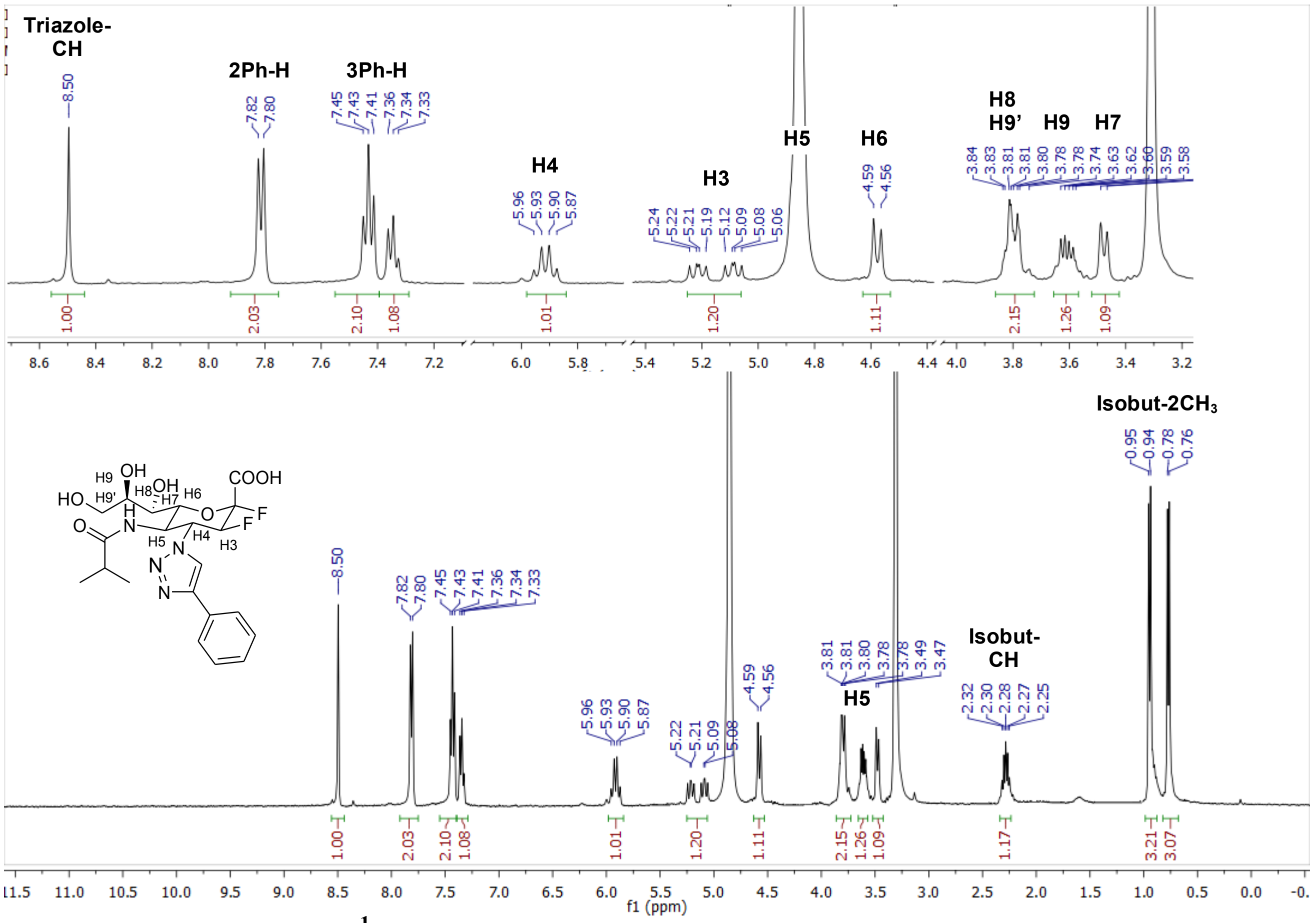

^{1}H NMR spectrum of 7 (400 MHz, CD_3OD)

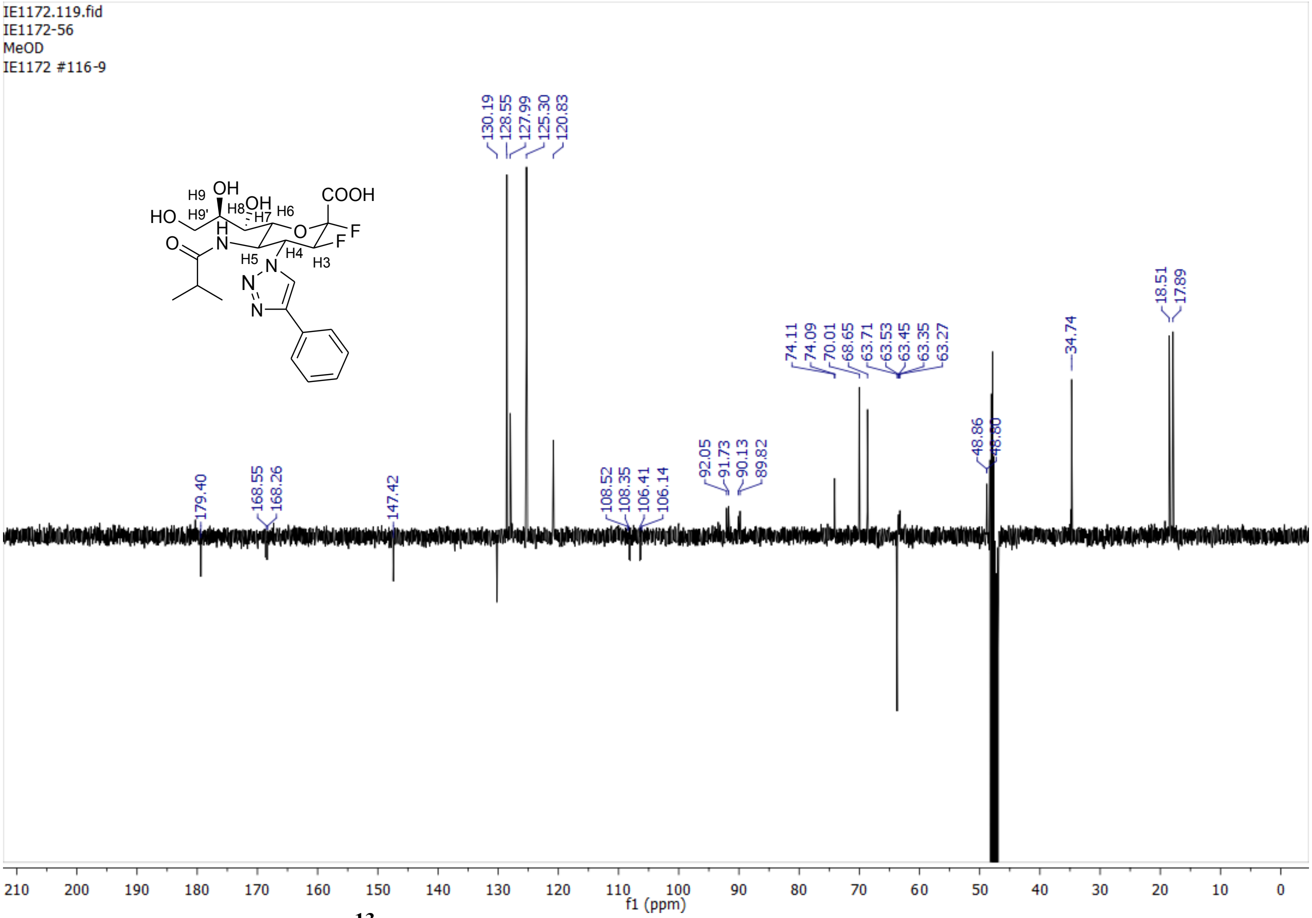

^{13}C NMR spectrum of 7 (100 MHz, CD_3OD)

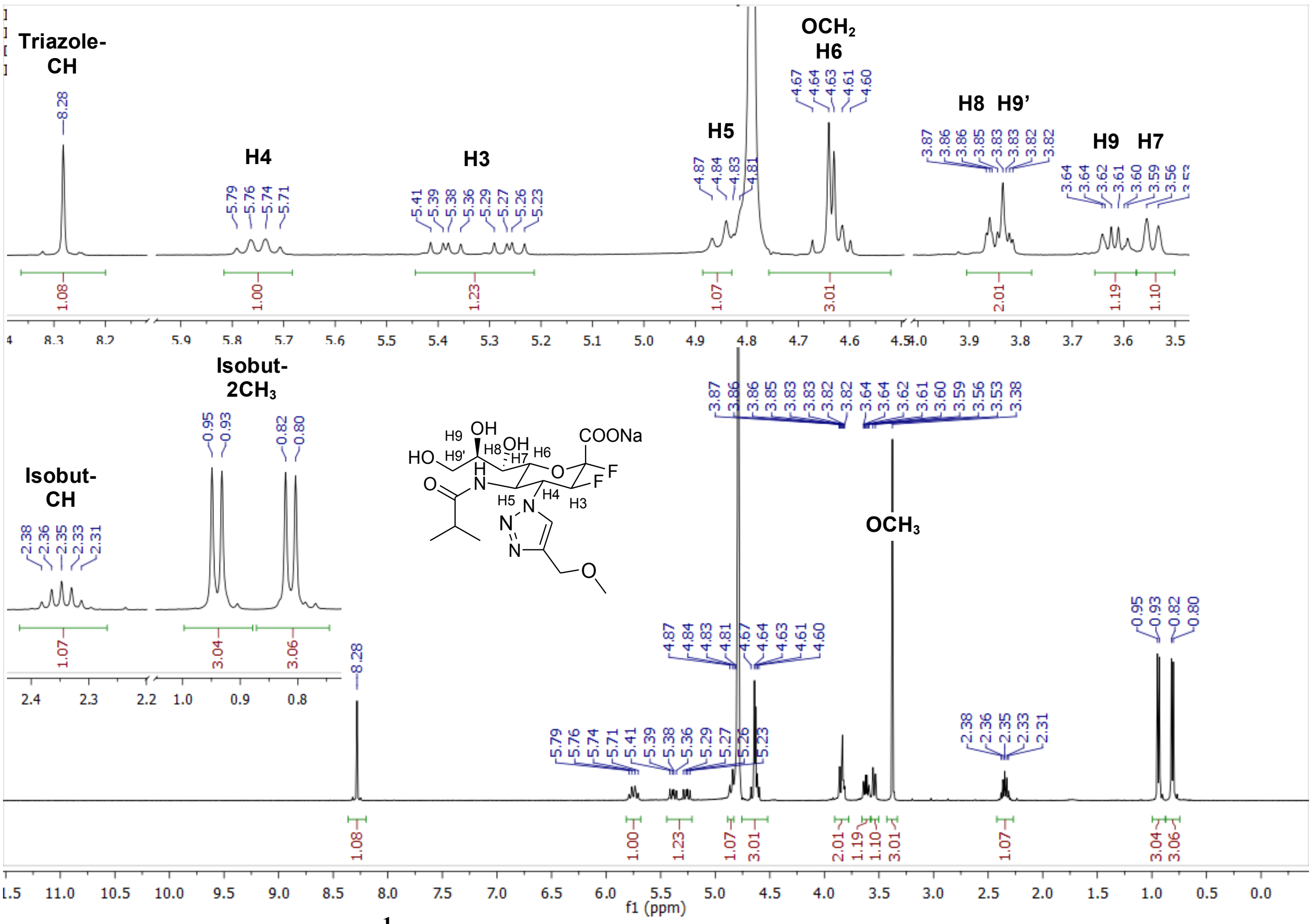

^{1}H NMR spectrum of 8 (400 MHz, D_2O)

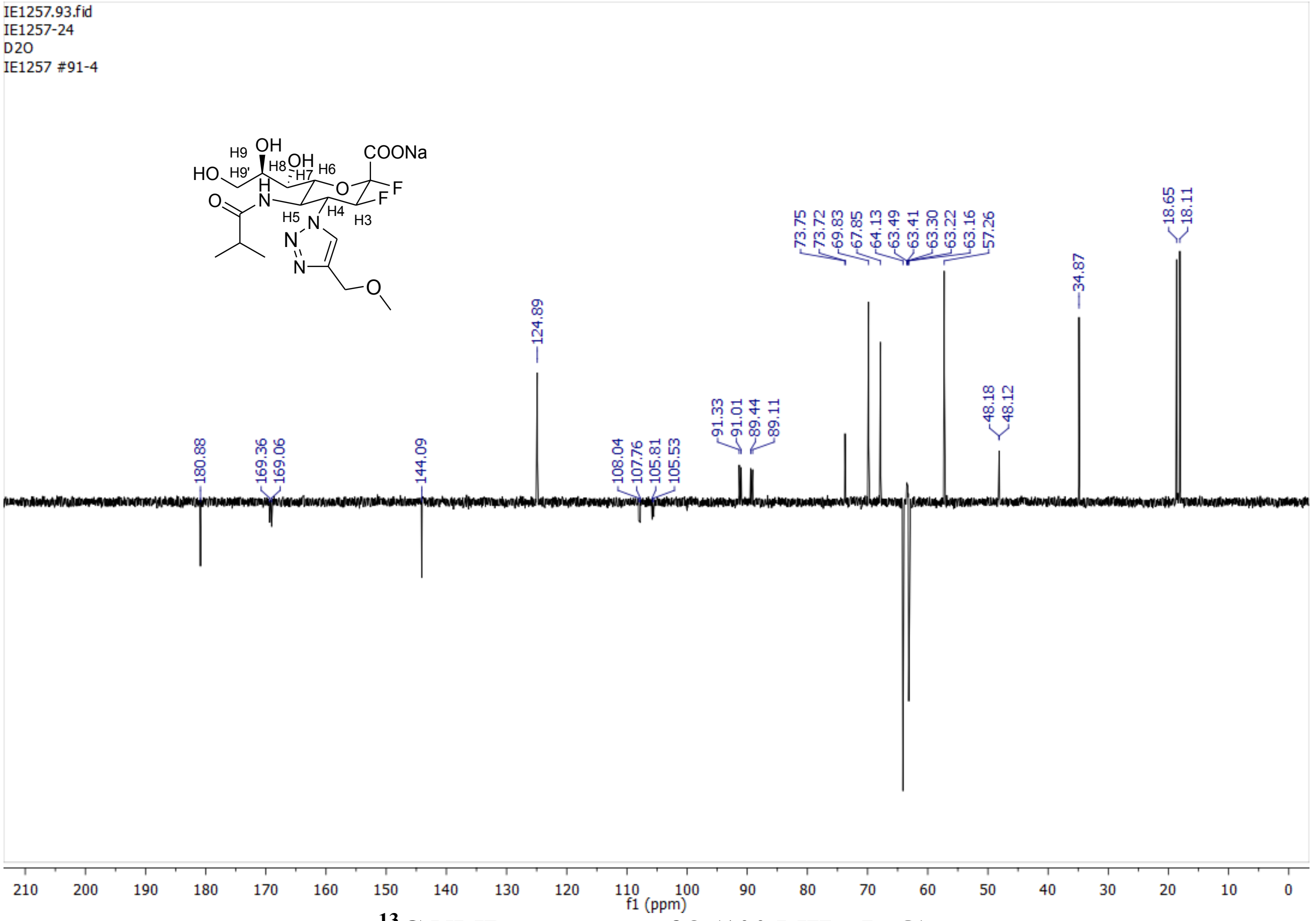

^{13}C NMR spectrum of 8 (100 MHz, D_2O)
